# Composition and Diversity of Natural Bacterial Communities in Mabisi, a Traditionally Fermented Milk

**DOI:** 10.3389/fmicb.2020.01816

**Published:** 2020-07-30

**Authors:** Himoonga Bernard Moonga, Sijmen E. Schoustra, Joost van den Heuvel, Anita R. Linnemann, Md Sainur Samad, John Shindano, Eddy J. Smid

**Affiliations:** ^1^Laboratory of Food Microbiology, Wageningen University and Research, Wageningen, Netherlands; ^2^Food Quality and Design, Wageningen University and Research, Wageningen, Netherlands; ^3^Laboratory of Genetics, Wageningen University and Research, Wageningen, Netherlands; ^4^Department of Food Science and Nutrition, School of Agricultural Sciences, University of Zambia, Lusaka, Zambia; ^5^Department of Microbial Ecology, Netherlands Institute of Ecology, Wageningen, Netherlands

**Keywords:** *Lactococcus*, lactic acid bacteria, selection pressure, firmicutes, tonga, barotse, microbial communities, Zambia

## Abstract

Many traditionally fermented milk products such as mabisi involve spontaneous fermentation, which can result in bacterial community composition variation due to selection pressure. The aim of this study was to determine the composition of bacterial communities in the different types of mabisi produced across Zambia and identify the factors that influence their composition. Samples of mabisi were collected across the country, and analyzed for pH and bacterial communities using 16S rRNA amplicon sequencing. We found that the bacterial community composition was dominated by members of two phyla, i.e., Firmicutes and Proteobacteria, from which the top 10 most abundant genera were *Lactococcus, Lactobacillus, Streptococcus, Enterobacter, Citrobacter, Klebsiella, Kluyvera, Buttiauxella*, *Aeromonas*, and *Acinetobacter*. The most dominant genus was *Lactococcus*, which was present in all types of mabisi produced from all regions. The mabisi products from traditional mabisi production regions (TMPRs) were dominated by lactic acid bacteria (LAB) whereas products from non-TMPRs were dominated by non-LAB species. Tonga mabisi, the most popular type of mabisi produced in non-TMPRs, had the most complex and diverse bacterial community composition compared to the other types, which included barotse, backslopping, creamy, and thick-tonga mabisi. Other factors that influenced bacterial community composition were geographical location, fermentation duration and pH while the type of fermentation container and producer did not. This study provides new insights that can be applied in starter culture development as well as microbial functionality studies.

## Introduction

A wide variety of ecosystems exist in nature that are dominated by microbial communities. It has been observed that similar systems harbor similar communities in terms of species diversity and that this is driven by selection due to both biotic and abiotic factors, a process that has also been named “species sorting” (Langenheder and Székely, 2011; Székely and Langenheder, 2014). While this process is widely acknowledged, little experimental work exists that addresses how selection can shape species communities. These include microbial communities in the soil, human gut as well as fermented foods (de Vries and Griffiths, 2018; Rowland et al., 2018; Anal, 2019). Fermented foods are natural environments of mixed communities of co-existing microbes.

Fermented milk products are popular around the world and are important for delivering nutrients, for providing beneficial microbes to promote a balanced gut microbiota and for imparting desirable organoleptic properties on foods (Sybesma et al., 2015; Singh et al., 2017; Anal, 2019). The common fermented dairy products on the world market include cheese, yogurt, kefir, and many others. Most of these products derive their recipes from artisanal or traditional processes that involve spontaneous fermentation by complex microbial communities (Smid, 2015). In Africa, many traditional fermented (dairy) products made at household level exist, whose recipes and production techniques are handed down from one generation to another, i.e., from mother to daughter or father to son. These production techniques vary from one region/country to another and this may have a bearing on the microbial composition of the respective products. To gain more insights into such products and to understand, if variation in production method or geographical location may impose a selective pressure leading to variations in microbial composition, we took mabisi as a case study.

Mabisi is a Zambian traditionally fermented milk made by spontaneous fermentation of raw milk at ambient temperature for 2 days or more (Schoustra et al., 2013; Moonga et al., 2019). This product is popular and widely consumed with the staple maize porridge (nshima) as well as other types of foods such as rice, sweet potatoes, pumpkins, and fruits (Moonga et al., 2019). Zambia is a large country (752,000 km^2^) with a population consisting of a variety ethnic groups [Central Statistical Office (CSO) and ICF International, 2014] and mabisi production is traditionally practiced in regions or provinces with high cattle population. However, the demand for the product has steadily been rising in the cities. A previous study by Moonga et al. (2019) has shown that there are seven production methods of mabisi: tonga, illa, backslopping, creamy, cooked, barotse, and thick-tonga spread around the country with tonga type being the most popular and widely practiced in all regions and by all ethnic groups. This study further highlights the key production parameters as being temperature, type of fermentation containers types, season, backslopping, and alternate whey removal and addition of raw milk. However, there are limited studies on the microbes involved in this spontaneous traditional fermentation.

The spontaneous fermentation of mabisi relies on microbes from the surrounding production environment: the raw milk, production utensils (containers and buckets), hands of producers and the air. A study by Schoustra et al. (2013) has shown that mabisi samples collected in the southern and central parts of Zambia contain 6–8 species of lactic acid bacteria (LAB) and acetic acid bacteria (AAB). However, that study was limited in the number of samples that were analyzed and did not cover all mabisi production regions representative of all variation in production methods in the country. As in all natural species communities in nature, the microbial communities in spontaneously fermented food products are shaped by both abiotic and biotic factors (Domínguez-Manzano et al., 2012; Bokulich et al., 2016). With the different production practices in the country, we hypothesize that the microbial community composition varies as a function of production region, producer, type of fermentation container, and type of mabisi. Studies across the African continent have shown variation in microbial composition of traditional fermented milk products from country to country (Akabanda et al., 2010; Osvik et al., 2013; Parker et al., 2018) but these have not been linked to variations in production practice or sampling location, which may exert key selection pressures on the microbial ecosystem impacting their species composition and dynamics. This motivated us to investigate the composition of the microbial communities of mabisi samples country-wide in Zambia using high-throughput DNA sequencing techniques.

In this study, we investigated the bacterial community composition of mabisi across the provinces of Zambia, and subsequently, identified the key factors that determine the anticipated diversity in bacterial community composition. We used culture independent methods and focused on the bacterial community composition as earlier work has shown that yeast was rarely detected in mabisi (Schoustra et al., 2013). We believe that this study will provide insights into the types of microbes that are involved in the fermentation of mabisi and the factors that shape their community structure. This information is important for understanding the ecology of these microbial communities and will form a basis for more fundamental research on how selective forces may affect microbial dynamics and functionality. More practically, it gives an overview of the potential candidate microbes to be used in starter culture development, which will be crucial for product optimization in order to meet the demand of both the rural and urban consumers.

## Materials and Methods

### Sample Collection

**FIGURE 1:**
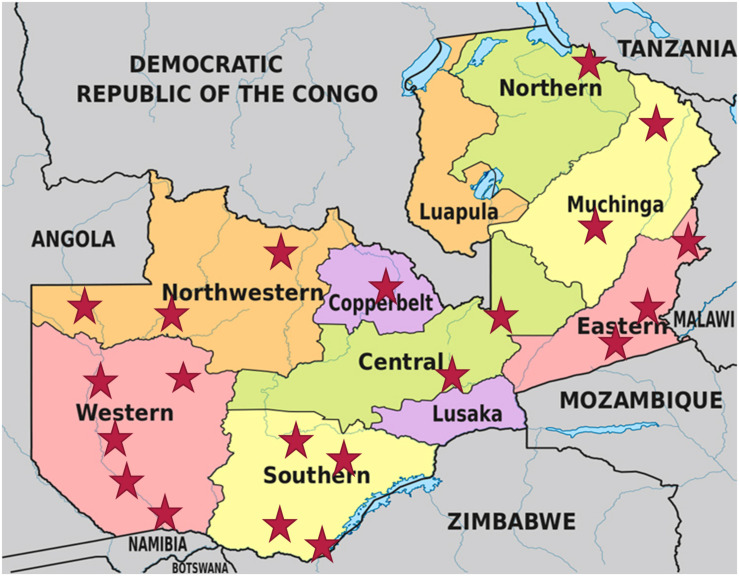
Map of Zambia showing all provinces and the sampling sites, which are denoted by the red stars. (Map adapted from https://www.researchgate.net/figure/Map-of-Zambia-showing-its-provinces-and-neighboring-countries_fig1_332258490).

A total of 168 mabisi samples (Table 1) were collected across eight provinces of Zambia between May and August 2016 (Figure 1).

**TABLE 1:** Number of mabisi samples collected from each sampling location.

**Sampling location**		**Number of samples**
Traditional mabisi	Western province	55
production region (TMPR)	Southern province	40
	Central province	11
Non-TMPR	Eastern province	25
	Copperbelt province	8
	North-western province	14
	Muchinga province	11
	Northern province	4
Total		168

The samples were collected during the dry and cold season of the year when milk production is low and as a result certain regions with fewer cattle farmers had fewer samples. The samples were collected from farmers, traders in the local markets and Milk Collection Centers (MCCs) of farmer Cooperatives. However, for the purpose of this study the latter two were both treated as traders. The farmers that provided samples were identified through their Cooperatives with the assistance of the personnel from the Ministry of Fisheries and Livestock (MFL) of the Zambian Government and were part of those interviewed during a survey on mabisi production practices conducted by Moonga et al. (2019). The following information was recorded for all samples collected: sampling location (province and district), production method, age of mabisi (fermentation duration in days), type of fermentation containers used (calabash, plastic or metal), and type of producer (farmers, traders or MCCs). The mabisi samples collected were produced using five production methods: tonga, backslopping, barotse, creamy, and thick-tonga mabisi reported by Moonga et al. (2019).

Mabisi samples were collected using 500 ml sterile plastic bottles which were immediately stored on ice in a cool box to stop the fermentation and transported to the laboratory where physicochemical analysis and DNA extraction were carried out. The samples were analyzed for pH and titratable acidity (TTA) upon arrival at the Food Chemistry Laboratory, Department of Food Science and Nutrition of the University of Zambia. The samples for microbial analysis were separated prior to these analyses. Bacterial community analysis was performed by culture independent techniques which, involved DNA extraction and high throughput 16S rRNA amplicon sequencing. To do this, mabisi samples were pipetted into 1.5 ml eppendorf tubes and centrifuged (12,000 rpm) for 2 min after which, the supernatant was poured out and the pellet was frozen at −20°C for subsequent DNA extraction.

### Physicochemical Properties

The pH for the mabisi samples was analyzed using a calibrated digital pH meter and the TTA was analyzed according to the AOAC official methods (AOAC, 2005).

### Amplicon Sequencing

The frozen mabisi sample pellets were thawed and the DNA was extracted and purified as described by Schoustra et al. (2013). The extracted DNA was subsequently, sent for bacterial 16S rRNA gene amplicon paired-end sequencing of the V4 hypervariable region (341F–785R) on the MiSeq Illumina platform performed by LGC genomics (Berlin, Germany).

For further data processing and statistics, the QIIME pipeline (Caporaso et al., 2010), modified by Bik et al. (2016) was used. Paired-end reads were joined using join_paired_ends.py (with minimum overlap 10 basepairs) after which sequences were trimmed and filtered using cutadapt [v1.11 –q 20, –m 400, (Martin, 2011)] using the known primer sequences CCTACGGGNGGCWGCAG and GACTACHVGGGTATCTAAKCC to trim both sides of the sequence. These trimmed sequences were then checked for chimera’s, using uchime [v4.2.20, gold database, (Edgar et al., 2014)], sequences with a lower chimera score than 0.28 were retained. The sequences were filtered by Qiime script (split_libraries_fastq.py, phred offset value: 33) and then clustered into Operational Taxonomic Units (OTUs) at 97% sequence similarity using the SILVA reference database (version 132; Quast et al., 2013) and UCLUST (Edgar, 2010) using “pick_open_reference_otus.py” Qiime script. For assigning taxonomic classification, BLAST analysis (with default *e*-value) was done against the SILVA database (Altschul et al., 1990; version 132). All downstream analysis were performed in R (R Development Core Team, 2008).

### Statistical and Data Analysis

The data was analyzed using one-way analysis of variance (ANOVA) at 95% significance level and mean comparisons were performed by Turkey test at 95% significance level using SPSS version 22. The relationship between bacterial community diversity, location and mabisi production methods was analyzed using non-metric multidimensional scaling (NMDS). Further, the analysis of similarities (ANOSIM) and permutational multivariate analysis of variance (Adonis test) were performed in R version 3.6.0 (R Development Core Team, 2008) using the phyloseq (McMurdie and Holmes, 2013) and vegan (Oksanen et al., 2019) packages to investigate the effect of different categorical variables (e.g., production method, fermentation container, and type of producer, geographical location, fermentation duration) on bacterial communities in mabisi. In addition, the Mantel test was performed to determine the correlation between bacterial community structure and pH. The bacteria community diversity was measured by the shannon index and richness.

## Results

**FIGURE 2:**
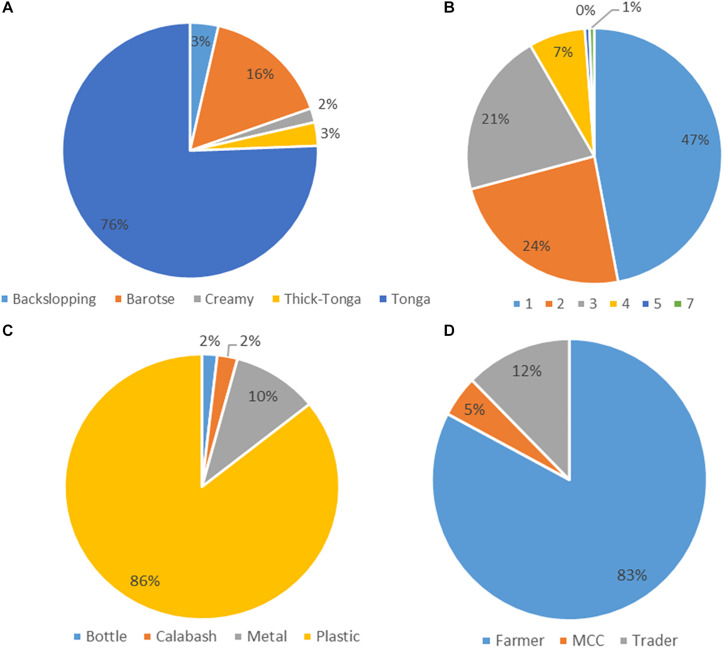
Frequency data of the production parameters: **(A)** Production method of mabisi, **(B)** fermentation time in days (age), **(C)** type of fermentation container, and **(D)** type of producer.

The mabisi samples were analyzed for physicochemical properties: pH and TTA, and for bacterial community composition using 16S rRNA amplicon sequencing. All the 168 mabisi samples collected around the country were classified according to different production factors identified: production method, duration of fermentation (age), type of fermentation containers used and type of producers (Figure 2). We found that most of the samples collected were produced using tonga-type production method whose final product is referred to as “tonga mabisi” (76%) and the least product type produced was “creamy mabisi” (2%). The most popular container used for fermentation was the plastic container (86%). A large proportion of the samples were produced by farmers (83%) and the commonest fermentation duration was 1 day (47%).

### Physicochemical Properties

**FIGURE 3:**
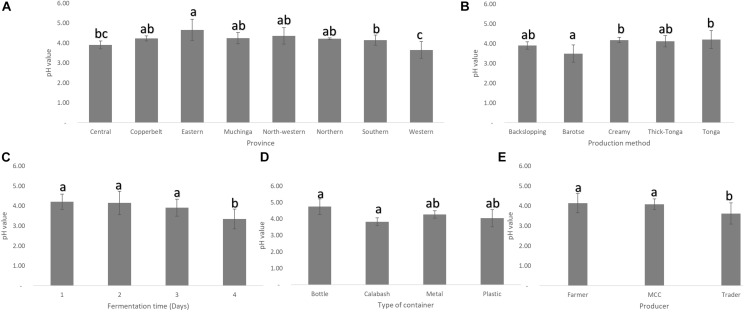
Mean pH of mabisi samples. pH of mabisi from different: **(A)** provinces, **(B)** production methods, **(C)** fermentation time, **(D)** fermentation container, and **(E)** producers. The bars with different letters for each mean pH value indicate statistically significant differences (*p* < 0.05).

Mabisi samples collected from Eastern province had the highest mean pH while those from Western province had the lowest, but both were significantly different from those from the rest of the other provinces (Figure 3A). In terms of the types of mabisi, barotse mabisi had a significantly lower mean pH than the rest. Backslopping mabisi also had a lower pH than tonga, creamy and thick-tonga mabisi but the difference was not significant (Figure 3B). The traders produced mabisi with lower mean pH compared to mabisi produced by the farmers or MCCs, while mabisi fermented in the calabashes had the lowest mean pH compared to mabisi fermented in either plastic or metal containers (Figures 3D,E). The mean pH of mabisi samples fermented for 1 day was the highest and the lowest pH values were observed in mabisi samples fermented for 4 days (Figure 3C).

### Bacterial Community Composition

**FIGURE 4:**
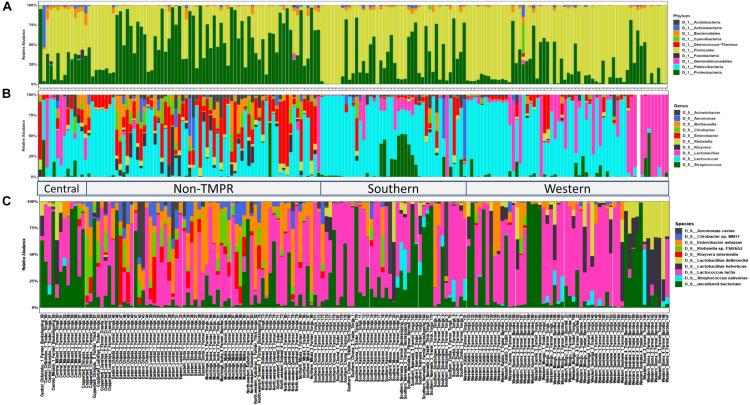
Bacterial community composition of the top 10 relative abundant bacteria in mabisi at the level of: **(A)** phylum, **(B)** genus, and **(C)** species. Each bar represents an individual sample. Two main regions are shown the traditional mabisi production region (TMPR) represented by Western, Southern and Central provinces, and the non-TMPR.

The bacterial community composition of mabisi was dominated by species belonging to the phyla, Firmicutes and Proteobacteria (Figure 4A). The other phyla that made up the top 10 most abundant bacteria include Acidobacteria, Actinobacteria, Bacteriodetes, Cyanobacteria, Fusobacteria, Patescibacteria, Deinococcus-thermus, and Gemmatimonadetes.

When the data of all sampled mabisi are combined, *Lactococcus* is the genus with the highest relative abundance. The other genera that make up the top 10 of most abundant genera include *Lactobacillus*, *Streptococcus*, Kluyvera, *Klebsiella*, *Enterobacter*, *Citrobacter*, *Buttiauxella*, *Aeromonas*, and *Acinetobacter* (Figure 4B). Mabisi is traditionally produced in Western, Southern and parts of Central province, which in this study can be collectively referred to as “Traditional mabisi production regions (TMPRs).” The bacterial community composition of mabisi from this region, was dominated by LAB of the genera *Lactococcus*, *Lactobacillus*, and *Streptococcus*. Within the TMPR, mabisi from Western province had a larger proportion of *Lactobacillus* than the rest as was the case with Southern Province mabisi for *Streptococcus*. Mabisi samples from the non-TMPR of Eastern, North-western, Muchinga, and Northern provinces had a more complex bacterial community composition collectively dominated by non-LAB, although *Lactococcus* was present in all samples. From the non-TMPR, only mabisi samples from Copperbelt province were dominated by *Lactococcus* but still had a high proportion of *Enterobacter*.

The top 10 most abundant species found in mabisi included *Lactococcus lactis*, *Streptococcus salivarius*, *Lactobacillus helveticus*, *Lactobacillus delbrueckii*, *Kluyvera intermedia*, *Klebsiella* sp. *Enterobacter asburiae*, *Citrobacter* sp., and *Aeromonas caviae* (Figure 4C). The top 20 and 30 most abundant species (Supplementary Figure S1) include some of LAB species reported by Schoustra et al. (2013) that are absent in the top 10 most abundant species.

### Bacterial Diversity

**FIGURE 5:**
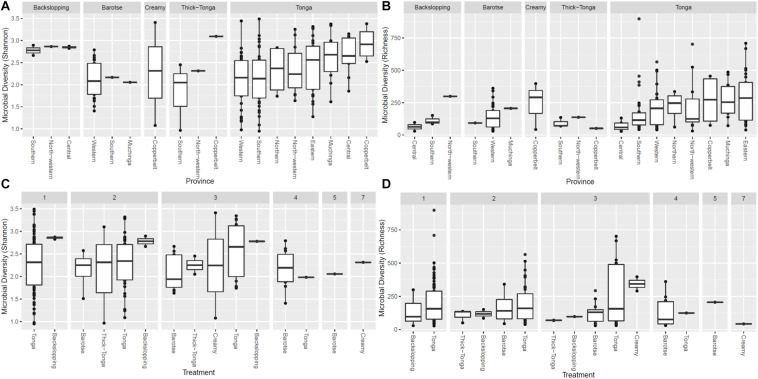
Bacterial alpha diversity of mabisi according to production method **(A)** Shannon index and **(B)** Richness and fermentation period **(C)** Shannon index and **(D)** Richness.

**FIGURE 6:**
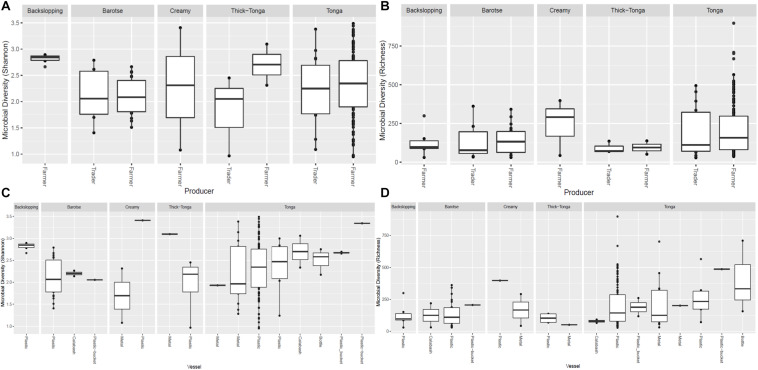
Bacteria diversity of mabisi in terms of producer **(A)** Shannon index and **(B)** Richness and type of container **(C)** Shannon index and **(D)** Richness.

The alpha diversity (i.e., the mean species diversity) of the mabisi bacteria community was analyzed by richness and Shannon index (Figures 5, 6). The results show that tonga mabisi was richer and more diverse in bacterial community composition than the other types of mabisi (Figures 5A,B). In addition, when we consider all tonga mabisi samples from all sampling sites (provinces), we observe that samples from the non-TMPR of Eastern, Muchinga, Northern, Copperbelt provinces were richer and more diverse than the TMPR of Western and Southern provinces (Figures 5A,B). Creamy mabisi was richer than backslopping, barotse, and thick-tonga mabisi but backslopping mabisi had a more diverse bacterial community composition than the other three (Figures 5A,B).

Tonga mabisi was richer in terms of diversity (no. of species) for samples that were fermented for 1, 2, and 3 days (Figure 5D) compared to those fermented for longer periods of time. Those fermented for 1 day showed more diversity than those fermented for 2 and 3 days (Figure 5C). Furthermore, tonga mabisi samples fermented for 1, 2, and 3 days were more diverse than the other types of mabisi.

The tonga mabisi samples were richer in bacterial community diversity than the other types of mabisi but there were no large differences in bacterial community diversity between samples produced by the farmers and the traders who sale their products in the local markets (Figure 6B). Tonga mabisi had more samples with higher diversity than the other types of mabisi, but only thick-tonga mabisi showed significant differences between the producers: the farmers produced mabisi with a higher diversity compared to the traders (Figure 6A). Tonga mabisi samples fermented in a glass bottle were richer in terms of bacterial community diversity than those fermented in plastic, metal and calabashes containers (Figure 6D). Both the bottle and the calabash fermented tonga mabisi samples had a slightly higher diversity as indicated by the shannon index than those fermented in metal and plastic containers (Figure 6C).

### Relationship Between Bacterial Community Diversity, Location and Mabisi Production Method

**FIGURE 7:**
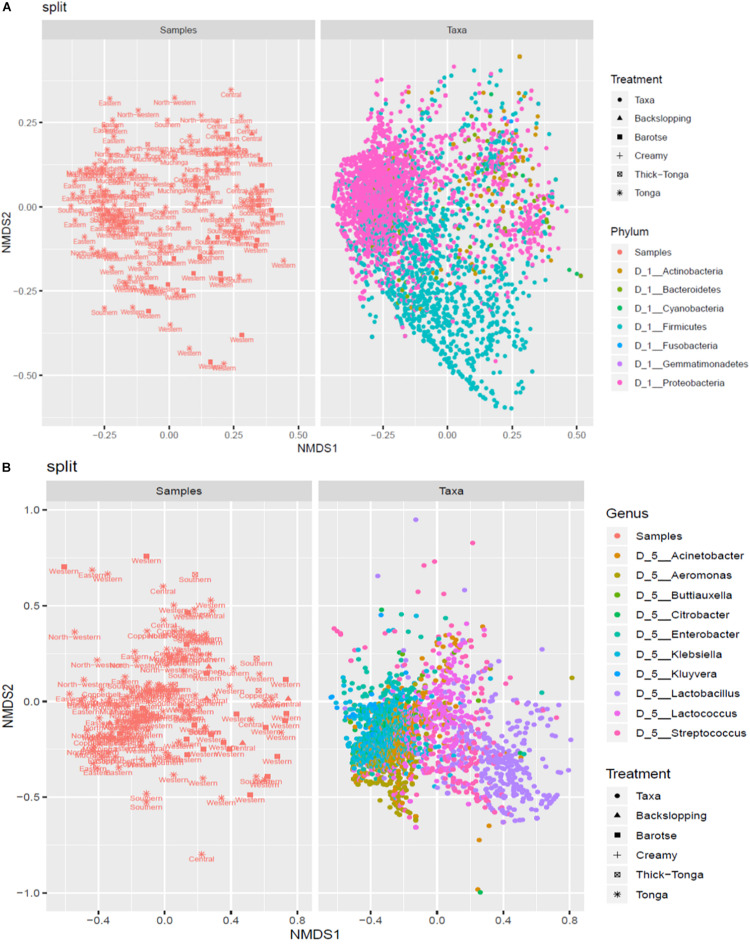
Non-metric multidimensional scaling (NMDS) plot of the relationship between taxa and location and production method at **(A)** phylum level and **(B)** genus level.

The mabisi samples were analyzed by NMDS to determine the association between type of mabisi/location and taxa. Figure 7A shows two main clusters of the phyla Firmicutes and Proteobacteria as well as a smaller one of Actinobacteria. The Firmicutes cluster is dominated by mabisi samples from Western and Southern provinces as well as the barotse and tonga types of mabisi whereas the Proteobacteria cluster is dominated by samples from the non-TMPRs and mostly, tonga type mabisi.

At genus level (Figure 7B), we were able to discriminate four big clusters dominated by *Lactobacillus*, *Lactococcus*, *Enterobacter*, and *Aeromonas*, and two smaller clusters dominated by *Streptococcus* and *Klebsiella*. The *Lactobacillus* cluster was associated with the barotse mabisi and samples mostly from Western with a few from Southern and Central provinces. *Lactococcus* had the largest cluster which included all the types of mabisi and provinces but was most prominent in the TMPR. The smaller *Streptococcus* cluster was associated with tonga, backslopping and barotse types of mabisi from the TMPR. The clusters of the non-LAB genera of *Aeromonas*, *Enterobacter*, and *Klebsiella* were mainly associated with tonga mabisi produced in the non-TMPR, however, other types of mabisi from the TMPR also had some samples with these bacteria at lower levels of abundance.

**FIGURE 8:**
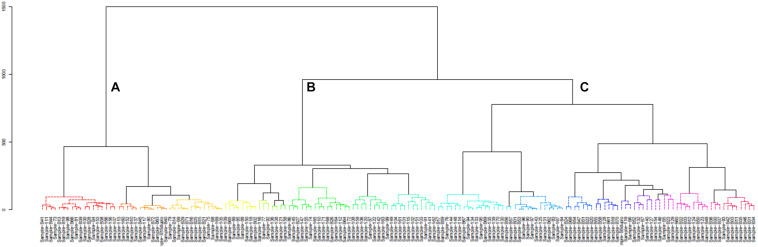
Cluster analysis of bacterial communities of all mabisi samples. The letters **(A–C)**, indicate the three main clusters.

A hierarchical cluster analysis of the bacterial communities in all mabisi samples was carried out and this resulted in three main clusters: A, B, and C (Figure 8). Cluster A was dominated by samples from TMPR (65%) while clusters B and C were populated mostly by samples from non-TMPR (>75%) and TMPR (>80%), respectively. In terms of types of mabisi, cluster A had tonga, barotse, and creamy mabisi, cluster B had mostly tonga mabisi and cluster C had all types: tonga, barotse, thick-tonga, creamy, and backslopping mabisi.

The effect of each factor on the bacterial community composition was analyzed by ANOSIM and Adonis test. We found that production method, geographical location (province and district) and fermentation duration all significantly influenced the bacterial community structure while the type of fermentation container and producer did not (Table 2). Furthermore, a mantel test showed that there was a significant correlation between pH and bacterial community structure (*r* = 0.2807, *p* < 0.001).

**TABLE 2:** ANOSIM and permutational MANOVA of categorical effects on bacterial communities in mabisi.

**Group**	**ANOSIM**		**ADONIS**	
	***R***	***p***	***R*^2^**	***p***
Production method/treatment (Tonga, Backslopping, Creamy, Thick–Tonga, Barotse)	0.158	0.001	0.045	0.001
Producer (Farmer, Trader)	0.068	0.068	0.017	0.009
Province (Location)	0.138	0.001	0.134	0.001
District (Location)	0.235	0.001	0.282	0.001
Vessel (Fermentation container)	0.007	0.453	0.026	0.232
Age (Fermentation duration)	0.112	0.001	0.023	0.001

## Discussion

The objective of this study was to determine the bacterial community composition of mabisi, its species diversity and the factors that influence community composition. The results show that mabisi is composed of bacteria from mainly two phyla (Firmicutes and Proteobacteria) with the most dominant LAB genera being *Lactococcus*, *Lactobacillus*, and *Streptococcus* and the non-LAB dominated by *Enterobacter*, *Aeromonas*, and *Klebsiella* (Figure 4). The LAB genera found in this study have also been reported by Schoustra et al. (2013) for fewer mabisi samples collected from two provinces only, Southern and Central provinces, which are both part of TMPR. These LAB species dominate the entire TMPR, which also includes the Western province. However, our findings also reveal some non-LAB genera (mostly Gram-negative species), which dominate the non-TMPR and some of them have been reported in other African traditional fermented milk (Osvik et al., 2013). The TMPR are the regions (Table 1) with more cattle and milk production (Musika, 2017; MFL, 2018; Moonga et al., 2019), and thus, the production of mabisi has a longer history and is carried out at a larger scale in these regions. In contrast, the non-TMPR are those regions with fewer cattle and low milk production, although Eastern province within this region has high cattle population but low milk production and consequently, low mabisi production as well. Moreover, mabisi consumption for some ethnic groups in this province is not common.

Interestingly, *Lactococcus* was the most dominant genus present in all mabisi samples and can be assumed to be driving the fermentation process. *Lactobacillus* was region and product specific, mostly found in the TMPR particularly, in barotse and backslopping mabisi, which were mainly produced by traders. *Lactobacillus* was more dominant in mabisi samples with low pH (pH < 4), which explains its dominance in mabisi samples from Western province as well as barotse and backslopping mabisi samples (Figure 3). This is mainly because *Lactobacillus* sp. are in general more acid tolerant than *Lactococcus* species (Axelsson, 2004). *Streptococcus* sp. were also found in the TMPR but had a high relative abundance in samples collected from Southern province particularly, from one district.

The top 10 species is made up of four LAB, five non-LAB and unclassified bacteria species (Figure 4). The most abundant species is *Lactococcus lactis* which is found in all mabisi samples. This species seems necessary for the fermentation to take place for all types of mabisi. It is a well-known homofermentative lactic acid bacterium used in many fermented dairy products like cheese and quark (Murtaza et al., 2014; Farkye, 2017). However, the different types of mabisi have different bacteria that are responsible for fermentation. For instance, tonga mabisi always has *Lactococcus* sp. especially for tonga mabisi from non-TMPR but the one from TMPR will in addition to *Lactococcus* species also have *Streptococcus salivarius* and in a few cases *Lactobacillus delbrueckii* and *Lactobacillus helveticus* which is also the case for thick-tonga mabisi. However, barotse and backslopping mabisi have all three genera present with *Lactobacillus* having a higher relative abundance than in the other types of mabisi. This suggests that for a well-designed mabisi starter culture, we should choose the right combination of LAB as well as take into account the specific production practices that may influence these bacteria in order to produce a product with desirable organoleptic properties. Microbes are known to have specific effects on organoleptic properties of dairy products (Leroy and De Vuyst, 2004; Lucey, 2004; Smid and Kleerebezem, 2014). Therefore, the effect of the different bacterial community composition on the organoleptic properties of mabisi requires further investigation. Further, with growing interest for artisanal products, this information can also be crucial in the design of “autochthonous” starter cultures able to mimic the spontaneous fermentation for the production of mabisi also referred to as the “third way” (Capozzi et al., 2020; Tamang et al., 2020). Connected to this, further work could specifically address the effect of variations in backslopping on the microbial community composition and associated product attributes.

In terms of bacterial community diversity, the mabisi produced in the TMPR was less diverse and was mostly dominated by the LAB genera compared to the ones produced in the non-TMPRs, which had a more complex and diverse composition. There was a relationship between the final pH and microbial diversity: the lower the pH, the lower the diversity of the bacterial community. This was particularly observed in samples from Western province, which were dominated by *Lactococcus* species and *Lactobacillus* species. Conversely, samples with a high pH exhibited a higher diversity as evidenced with samples from Eastern province. The latter province had mabisi with the highest mean pH probably because most of the farmers that supplied the samples rarely made mabisi due to the low amounts of milk produced during the dry season which was often consumed fresh. Moreover, it is not cultural for some ethnic groups in this province to make mabisi. It should also be pointed out that spontaneous fermentation can pose potential microbiological risks (Capozzi et al., 2017). In this study, mabisi with high pH (pH above 4.5) especially from non-TMPRs would pose the higher risk. Verifying this would require further study. However, our discussions with processors and consumers as part of sample collection for this study did not reveal cases of illness associated with mabisi consumption.

The bacterial community composition results, also show a clear signature of the type of selection pressure acting on the fermenting milk. In this case, we consider mabisi as the ecosystem which is being subjected to selection pressures in the form of production practices, geographical location, use of specific fermentation containers, influence of handling by producers and fermentation duration. This study has demonstrated that geographical location has a significant effect (*P* < 0.001) on the bacterial community composition as shown by the difference in bacterial community structure for the two main regions that have been identified: TMPR and non-TMPR. Non-TMPR samples exhibited a more complex community probably due to low scale and frequency of production, mainly limited to one production method whereas the communities in mabisi samples from TMPRs were less complex and mainly dominated by LAB. This may be due to more production methods used, more frequent and larger scale of production of mabisi in this region may have led to prolonged co-culturing of LAB that has resulted in this particular outcome.

The production practices may have also contributed to shifts in bacterial community compositions, tonga type production method is a batch production of 1–3 days and the product is usually consumed within that period. The barotse type method involves the alternate removal of whey and addition of raw milk, and takes 4–7 days to produce (Moonga et al., 2019). On the other hand, backslopping type method involves using a portion of a batch of mabisi as starter for the next one and this backslopping process can go on for several cycles. The results obtained in this study show that there are differences in the bacterial community composition of these products (Figure 7B). Tonga mabisi is dominated by *Lactococcus* species and other non-LAB genera while barotse mabisi is dominated by more LAB genera, particularly, *Lactococcus* species, and *Lactobacillus* species, and backslopping mabisi in addition has *Streptococcus* species. Thick-tonga and creamy mabisi were similar to tonga mabisi from TMPRs in bacterial community composition. Although, there were fewer samples for some types of mabisi, the statistical analysis shows significant differences (*P* < 0.001). Thus, it is clear that each production method exerts a certain selection pressure on the bacterial communities of mabisi, which results in different community composition. Formal experiments on bacterial community composition of different production methods of mabisi support these findings (Moonga, 2019).

The impact of the origin of the mabisi samples, being either directly from producers (farmers) or from traders, on the bacterial community composition was observed in the slightly higher richness and diversity of the bacterial community in samples collected from farmers compared to those collected from traders but was not significant (Figures 6A,B). A comparison of the OTUs of mabisi produced in different fermentation containers also did not have a significant effect on the bacterial community composition but fermentation duration did. The latter may influence the final pH of mabisi which also had a significant effect on the bacterial community composition. Unfortunately, the sampling did not give equal numbers of samples for each product type, producer, location, container, or fermentation duration. This means that to be able to interrogate this further, specific experiments with controls need to be carried out to give more substantive results on each factor. It should also be noted that the samples that were collected were endpoint samples of mabisi fermentation at different stages, which were interpreted based on information provided by the producers on their respective samples.

In conclusion, the mabisi bacterial community is dominated by four LAB and five non-LAB genera. The bacterial composition is more diverse for mabisi samples collected in non-TMPR than TMPR with longer production history and wider variety of the types of mabisi produced. The non-TMPR primarily produced tonga mabisi dominated with *Lactococcus* and non-LAB species whereas the TMPR were dominated by *Lactococcus*, *Lactobacillus*, and *Streptococcus* with a much smaller proportion of non-LAB species. Therefore, the development of any mabisi starter culture would require selection of strains from these LAB species for specific types of mabisi products.

The geographical location, production method, fermentation duration and pH exerted significant selection pressures on the microbes in mabisi that shaped the outcome of the microbial community structure.

However, it is imperative to carry out experiments to ascertain the effect of each of these factors and determine the optimal production process conditions since this product is already on the market and as more MCCs are being established, standardized production protocols will ensure the production of consistent and good quality mabisi that meet the consumer needs.

## Data Availability Statement

The original contributions presented in the study are publicly available. This data can be found here: http://www.ncbi.nlm.nih.gov/bioproject/647247.

## Author Contributions

HM: conceptualization, data curation, formal analysis, funding acquisition, investigation, methodology, validation, visualization, roles/writing – original draft, and writing – review and editing. SS and ES: conceptualization, funding acquisition, methodology, project administration, resources, software, supervision, and writing – review and editing. JH: data curation, software, validation, and writing – review and editing. AL: conceptualization, funding acquisition, methodology, resources, supervision, and writing – review and editing. MS: data curation, formal analysis, software, validation, visualization, and writing – review and editing. JS: conceptualization, funding acquisition, methodology, project administration, resources, supervision, and writing – review and editing. All authors contributed to the article and approved the submitted version.

## Conflict of Interest

The authors declare that the research was conducted in the absence of any commercial or financial relationships that could be construed as a potential conflict of interest.

## Supplementary Material

FIGURE S1 The top 20 **(A)** and 30 **(B)** most abundant bacterial community species.
