# Supplementary figures and images for: Composition and Diversity of Natural Bacterial Communities in Mabisi, a Traditionally Fermented Milk
Source: Front Microbiol. 2020 Jul 30;11:1816. doi: 10.3389/fmicb.2020.01816 (PMC7406715; doi:10.3389/fmicb.2020.01816)

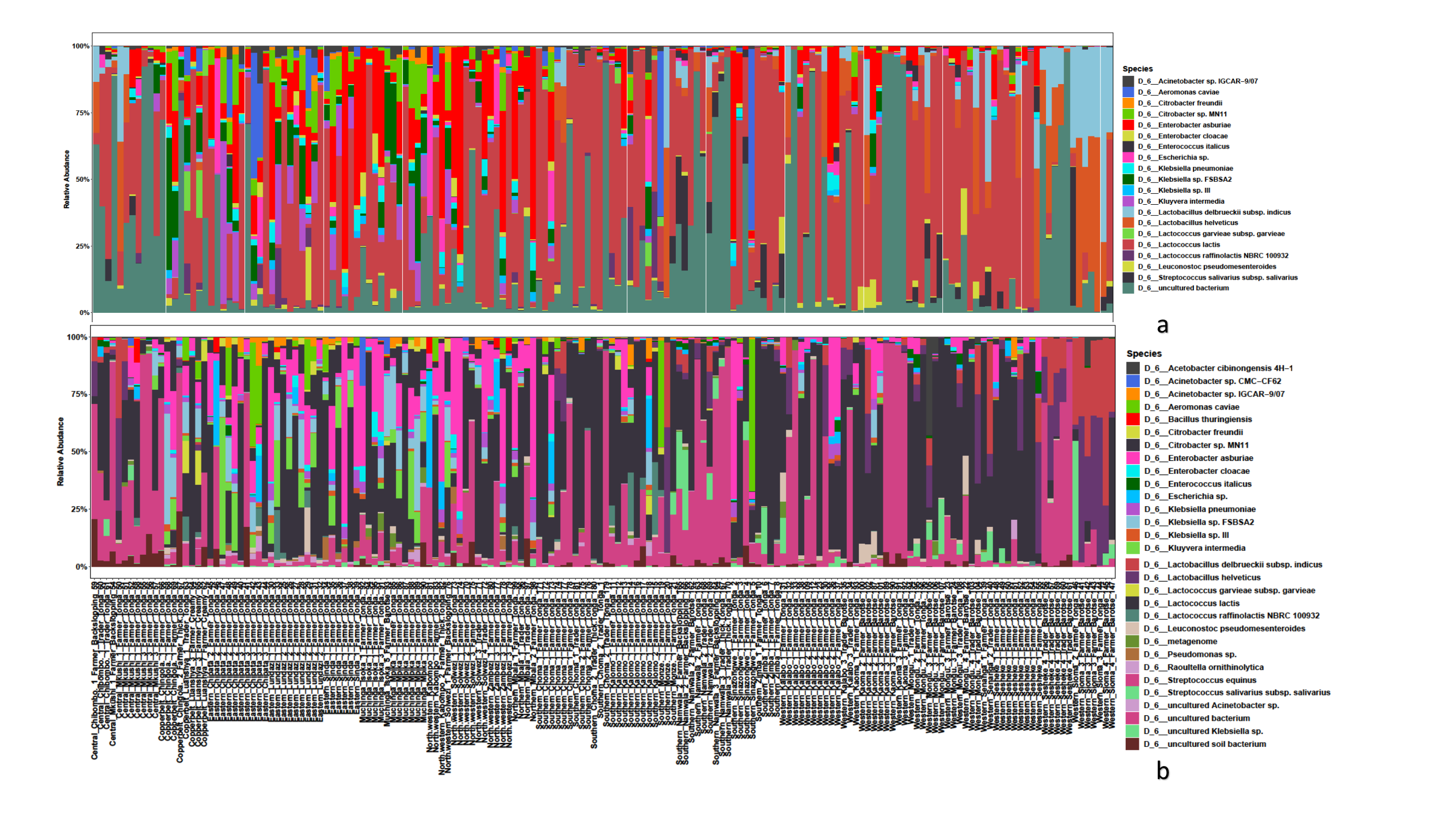

Supplement: FIGURE S1 — The top 20 (A) and 30 (B) most abundant bacterial community species. [file Image_1.tif]
